# Supplementary material for: Recyclable CRISPR/Cas9-mediated gene disruption and deletions in Histoplasma
Source: mSphere. 2023 Oct 11;8(6):e00370-23. doi: 10.1128/msphere.00370-23 (PMC10732100; doi:10.1128/msphere.00370-23)
Supplement: Supplemental material — Supplemental methods, tables, figures, and references. [file msphere.00370-23-s0001.docx]

**Supplemental material**

**Supplemental Materials and Methods**

**Cloning of CRISPR/Cas9 vectors.**

**Generation of CRISPR/Cas9 vector targeting *RYP2*.** The gRNA cassette targeting *RYP2* is based on the work of Nødvig *et. al.* (1) and was amplified in two parts with primer pairs OAS6208/OAS6048 for the 5’-fragment and OAS6049/OAS5708 for the 3’-fragment from pPTS608-Cas9-hyg-*PRA1* (2). Primers OAS6048 and OAS6049 have overlapping sequences for fusion PCR and are encoding the respective protospacer for *RYP2* as well as an inverted repeat of the first six base pairs of the protospacer, which precedes the hammerhead ribozyme sequence and ensures correct folding of the larger gRNA transcript. Both fragments were fused via fusion PCR with primers OAS5769/OAS5770, which add attB1 and attB2 sites for subsequent Gateway cloning. The fusion product was cloned into pDONR via standard Gateway BP-reaction protocol resulting in pBJ263. pBJ263 was recombined with pBJ219 via Gateway LR reaction leading to the final *RYP1* targeting CRISPR vector pBJ265.

**Generation of CRISPR/Cas9 vectors targeting *SRE1*.** For the disruption of *SRE1*, four different gRNA-constructs were created based on the same cloning concept, which was used for *RYP2*. First, 5’- and 3’-fragments were amplified from pPTS608-Cas9-hyg-*PRA1* with primer pairs OAS6208/OAS5715 & OAS5716/OAS5708 (*SRE1* gRNA-1), OAS6208/OAS5799 & OAS5800/OAS5708 (*SRE1* gRNA-2), OAS6208/OAS5801 & OAS5802/OAS5708 (*SRE1* gRNA-3) and OAS6208/OAS5803 & OAS5804/OAS5708 (*SRE1* gRNA-4). The corresponding gRNA fragments were joined via fusion PCR with primers OAS5769/OAS5770 and subsequently cloned into pDONR via BP-Gateway cloning, resulting in ENTRY vectors pBJ225 (*SRE1* gRNA-1), pBJ240 (*SRE1* gRNA-2), pBJ241 (*SRE1* gRNA-3) and pBJ242 (*SRE1* gRNA-3). The ENTRY vectors were recombined with the Cas9 destination vector pBJ219, leading to *SRE1* targeting CRISPR vectors pBJ230 (*SRE1* gRNA-1), pBJ243 (*SRE1* gRNA-2), pBJ244 (*SRE1* gRNA-3) and pBJ245 (*SRE1* gRNA-4).

**Generation of CRISPR/Cas9 vectors targeting *SID1*.** For the gRNA cassette targeting *SID1*, 5’- and 3’-fragments were amplified from pPTS608-Cas9-hyg-*PRA1* with primer pairs OAS6208/OAS6193 and OAS6194/OAS5708 and subsequently fused with primers OAS5769/OAS5770. The resulting gRNA-cassette was cloned into pDONR, resulting in pBJ268, which then got recombined with pBJ219 to generate *SID1*-targeting CRISPR vector pBJ270.

**Complementation vectors for *SRE1*.** The *SRE1* disruption mutant was complemented with episomal complementation vectors under either a native or constitutive promoter. For the native expression vector, the *SRE1* ORF including 1500bp of the 5’- promoter region was amplified from gDNA with primers OAS6201/OAS6202, which introduced attB-sites for Gateway cloning. The resulting fragment was cloned into pDONR via the Gateway system leading to pBJ272 and ultimately recombined with the expression vector pSB203, resulting in pBJ273. For the constitutive expression construct, the *SRE1* ORF was amplified from gDNA with primers OAS6325/OAS6202 and cloned via BP Gateway reaction into pDONR, resulting in pBJ279. pBJ279 was ultimately recombined with pSB234 (3, 4) in a LR Gateway reaction leading to pBJ280.

For transformation into *Histoplasma*, episomal vectors were digested with *Pac*I to excise the KanR cassette and expose telomeric sequences, which facilitate episomal maintenance. Approximately 10 ng of linearized plasmids were transformed into G217B *ura5^-^* via electroporation and transformants were selected on HMM agarose plates.

**Generation of dual sgRNA CRISPR/Cas9 vector for the deletion of *VEA1*.** To construct the first sgRNA cassette, 5’- and 3’- fragments containing a protospacer sequence targeting the 5’ end of the *VEA1* CDS were PCR amplified from pPTS608-Cas9-hyg-PRA1 using primer sets OAS6213/OAS6464 and OAS6463/OAS5708 respectively. Primer OAS6213 added part 1 of a *Sfr*I restriction site containing the spacer sequence. 5’ and 3’ fragments were joined by overlap extension PCR with primer set OAS6214/OAS5770 adding part 2 of the *Sfr*I-containing spacer as well as attB flanking sites. The complete sgRNA cassette was then cloned into pDONR/Zeo using Gateway cloning to produce plasmid pKW103. Finally, the sgRNA cassette was then recombined with destination vector pBJ219 to produce pKW104. The second sgRNA cassette targets the 3’ end of the *VEA1* CDS and does not include attB flanking sites. The 5’ and 3’ segments were PCR amplified from pBJ282 using primer sets OAS6381/OAS6458 and OAS5708/OAS6457 and joined by overlap extension PCR using primer set OAS6380/OAS6329. Cloning the second sgRNA cassette into the *Sfr*I-site of pKW104 by Gibson cloning produced the final plasmid pKW105.

**Supplemental Tables**

**Table S1: Plasmids used in this study**

| **Plasmid** | **Description** | **Reference** |
| --- | --- | --- |
| pDONR | Donor vector for Gateway cloning system; Zeo^R^ | Invitrogen |
| pSB23 | Gateway cloning vector for episomal RNAi | (5) |
| pSB203 | Gateway destination vector for expression under native promoter with *URA5*-marker | This study? |
| pSB234 | Gateway destination vector for overexpression under *GAPDH* promoter with *URA5*-marker | (3, 4) |
| pPTS608-Cas9-hyg | Binary vector for Agrobacterium mediated transformation containing codon optimized *cas9* sequence | (2) |
| pPTS608-Cas9-hyg-PRA1 | Binary vector for Agrobacterium mediated transformation containing codon optimized *cas9* sequence and sgRNA cassette targeting *PRA1* in *Blastomyces dermatitidis* | (2) |
| pBJ209 | pSB23 backbone +ApaI/NheI-site | This study |
| pBJ213 | pBJ209 + Gateway cassette in ClaI site | This study |
| pBJ219 | Episomal CRISPR/Cas9 vector with *ccdB* containing Gateway cassette for recombination with the gRNA cassette | This study |
| pBJ263 | pDONR with *RYP2* gRNA cassette | This study |
| pBJ265 | Episomal CRISPR/Cas9 vector targeting *RYP2* | This study |
| pBJ225 | pDONR with *SRE1* gRNA cassette 1 | This study |
| pBJ240 | pDONR with *SRE1* gRNA cassette 2 | This study |
| pBJ241 | pDONR with *SRE1* gRNA cassette 3 | This study |
| pBJ242 | pDONR with *SRE1* gRNA cassette 4 | This study |
| pBJ230 | Episomal CRISPR/Cas9 vector targeting *SRE1* with gRNA-1 | This study |
| pBJ243 | Episomal CRISPR/Cas9 vector targeting *SRE1* with gRNA-2 | This study |
| pBJ244 | Episomal CRISPR/Cas9 vector targeting *SRE1* with gRNA-3 | This study |
| pBJ245 | Episomal CRISPR/Cas9 vector targeting *SRE1* with gRNA-4 | This study |
| pBJ268 | pDONR with *SID1* gRNA cassette | This study |
| pBJ270 | Episomal CRISPR/Cas9 vector targeting *SID1* | This study |
| pBJ272 | pDONR containing *SRE1* + 1.5kb 5’promoter region | This study |
| pBJ273 | Episomal native promoter *SRE1* complementation vector | This study |
| pBJ279 | pDONR containing *SRE1* ORF | This study |
| pBJ280 | Episomal *SRE1* complementation vector under constitutive *GAPDH* promoter | This study |
| pKW103 | pDONR with *VEA1* 5’-end sgRNA cassette | This study |
| pKW104 | Episomal CRISPR/Cas9 vector targeting *VEA1* 5’-end | This study |
| pKW105 | Episomal CRISPR/Cas9 vector targeting *VEA1* 5’-end and 3’-end with two separate sgRNAs | This study |
| pBJ292 | Episomal CRISPR/Cas9 vector containing *P_gpdA_*::mCherry sgRNA1 | This study |
| pNA15 | Episomal CRISPR/Cas9 vector containing P*_GAPDH_*::mCherry sgRNA | This study |

**­**

**Table S2: Primers used in this study**

| **Primer** | **Sequence** | **Feature** |
| --- | --- | --- |
| OAS5744 | TGTAGGGCCCCCTGTGATTTGGTTGTTTGATTT | fw primer for pSB23 backbone adding ApaI-site |
| OAS5745 | TCCTGCTAGCATGGTATGAGGTTTGAGGCG | rv primer for pSB23 backbone adding NheI-site |
| OAS5734 | TCCGGATCGATACAAGTTTGTACAAAAAAGCTGAAC | fw primer for Gateway cassette including ClaI-site |
| OAS5735 | AGGATATCGATACCACTTTGTACAAGAAAGCTGA | rv primer for Gateway cassette including ClaI-site |
| OAS5736 | GGGGACAAGTTTGTACAAAAAAGCAGGCTTCGCTAGCTCAGACCTTGCGCTTCTTCT | fw primer for *cas9* incl. NheI- and attB sites |
| OAS5737 | GGGGACCACTTTGTACAAGAAAGCTGGGTAGGGCCCATGGACAAGAAGTATAGCATCGG | rv primer for *cas9* incl. ApaI- and attB sites |
| OAS6208 | GCGTAAGCTCCCTAATTGGC | fw primer for gRNA cassette |
| OAS6048 | GACGAGCTTACTCGTTTCGTCCTCACGGACTCATCAGACAGGGCGGTGATGTCTGCTCAAGC | rv primer for 5' fragment of CRISPR sgRNA cassette 1 for RYP2 disruption |
| OAS6049 | GGACGAAACGAGTAAGCTCGTCACAGGGTCTTCAGCAAATGTGTTTTAGAGCTAGAAATAGCAAG | fw primer for 3' fragment of CRISPR sgRNA cassette 1 for RYP2 disruption |
| OAS5708 | GAGCCAAGAGCGGATTCCT | rv primer of (t)*trpC* of sgRNA cassette |
| OAS5769 | GGGGACAAGTTTGTACAAAAAAGCAGGCTGCGTAAGCTCCCTAATTGGC | fw primer for sgRNA CRISPR-cassette incl. attB1-site |
| OAS5770 | GGGGACCACTTTGTACAAGAAAGCTGGGTGAGCCAAGAGCGGATTCCT | rv primer for sgRNA CRISPR-cassette incl. attB2-site |
| OAS5715 | GACGAGCTTACTCGTTTCGTCCTCACGGACTCATCAGCTCGGCCGGTGATGTCTGCTCAAGC | rv primer for 5' fragment of CRISPR sgRNA cassette 1 for SRE1 disruption |
| OAS5716 | GGACGAAACGAGTAAGCTCGTCCTCGGCAGACGGCTGCCGCAGTTTTAGAGCTAGAAATAGCAAG | fw primer for 3' fragment of CRISPR sgRNA cassette 1 for SRE1 disruption |
| OAS5799 | GACGAGCTTACTCGTTTCGTCCTCACGGACTCATCAGGGGCGCCGGTGATGTCTGCTCAAGC | rv primer for 5' fragment of CRISPR sgRNA cassette 2 for SRE1 disruption |
| OAS5800 | GGACGAAACGAGTAAGCTCGTCGGGCGCAAGGTCTCCTACAAGTTTTAGAGCTAGAAATAGCAAG | fw primer for 3' fragment of CRISPR sgRNA cassette 2 for SRE1 disruption |
| OAS5801 | GACGAGCTTACTCGTTTCGTCCTCACGGACTCATCAGATTATCCGGTGATGTCTGCTCAAGC | rv primer for 5' fragment of CRISPR sgRNA cassette 3 for SRE1 disruption |
| OAS5802 | GGACGAAACGAGTAAGCTCGTCATTATCTCGTCCACCTCGTGGTTTTAGAGCTAGAAATAGCAAG | fw primer for 3' fragment of CRISPR sgRNA cassette 3 for SRE1 disruption |
| OAS5803 | GACGAGCTTACTCGTTTCGTCCTCACGGACTCATCAGCTGGTACGGTGATGTCTGCTCAAGC | rv primer for 5' fragment of CRISPR sgRNA cassette 4 for SRE1 disruption |
| OAS5804 | GGACGAAACGAGTAAGCTCGTCCTGGTATCTTCCGCACGAGGGTTTTAGAGCTAGAAATAGCAAG | fw primer for 3' fragment of CRISPR sgRNA cassette 4 for SRE1 disruption |
| OAS6193 | GACGAGCTTACTCGTTTCGTCCTCACGGACTCATCAGTCGGTGCGGTGATGTCTGCTCAAGC | rv primer for 5' fragment of CRISPR sgRNA cassette for SID1 disruption |
| OAS6194 | GGACGAAACGAGTAAGCTCGTCTCGGTGATCTTGCGAGAGAAGTTTTAGAGCTAGAAATAGCAAG | fw primer for 3' fragment of CRISPR sgRNA cassette for SID1 disruption |
| OAS5782 | CTACCTTCAGAACTCATTGACCG | fw primer for *SRE1* CRISPR/Cas9 disruption fragment |
| OAS5783 | GGGATTTCTTCGACGACGT | rv primer for *SRE1* CRISPR/Cas9 disruption fragment |
| OAS6201 | GGGGACAAGTTTGTACAAAAAAGCAGGCTTTGACATTCACACATACGTATGTAC | fw primer for SRE1 5'UTR incl. attB1-site |
| OAS6202 | GGGGACCACTTTGTACAAGAAAGCTGGGTCTACTCCAACAACGCCAAC | rv primer for SRE1 ORF incl. attB2-site |
| OAS6325 | GGGGACAAGTTTGTACAAAAAAGCAGGCTATGACGGGCCTGCTG | fw primer for SRE1 incl. attB1-site |
| OAS6463 | GGACGAAACGAGTAAGCTCGTCTATGCTCAGTCTCATTCGGAGTTTTAGAGCTAGAAATAGCAAG | fw primer for vea1 protospacer 1 CRISPR sgRNA cassette |
| OAS6464 | GACGAGCTTACTCGTTTCGTCCTCACGGACTCATCAGTATGCTCGGTGATGTCTGCTCAAGC | rv primer for vea1 protospacer 1 CRISPR sgRNA cassette |
| OAS6457 | GGACGAAACGAGTAAGCTCGTCGGTAACAAATCCACATCGCGGTTTTAGAGCTAGAAATAGCAAG | fw primer for vea1 protospacer 2 CRISPR sgRNA cassette |
| OAS6458 | GACGAGCTTACTCGTTTCGTCCTCACGGACTCATCAGGGTAACCGGTGATGTCTGCTCAAGC | rv primer for vea1 protospacer 2 CRISPR sgRNA cassette |
| OAS6213 | AACTTGTTGCGTTCCTAGCCGCTATATTTGTCTCTTTGCGCGTAAGCTCCCTAATTGGC | fw primer for 5' fragment of CRISPR sgRNA cassettes with spacer region part 1 |
| OAS5708 | GAGCCAAGAGCGGATTCCT | rv primer for 3' fragment of CRISPR sgRNA casette |
| OAS6214 | GGGGACAAGTTTGTACAAAAAAGCAGGCTGCCCGGGCTAACTTGTTGCGTTCCTAGCC | fw primer for 5' fragment of CRISPR sgRNA cassettes with spacer region part 2 |
| OAS5770 | GGGGACCACTTTGTACAAGAAAGCTGGGTGAGCCAAGAGCGGATTCCT | rv primer for Fusion-PCR of sgRNA CRISPR-cassette incl. attB2-site |
| OAS6380 | AAAAGCAGGCTGCCCGCCCGGGCTAACTTGTTG | fw primer for Vea1 gRNA 1 incl. SrfI-spacer and pBJ292 overhang |
| OAS6329 | CGCAACAAGTTAGCCCGAGCCAAGAGCGGATTCCT | rv primer for gRNA cassette incl 16bp overhang for pBJ283-SrfI |
| OAS6659 | CATCCGCTCATGGTCTCCTC | fw Southern Blot Probe for Vea1 5' UTR |
| OAS6660 | CTGGTACGCAAACAATGCCC | rv Southern Blot Probe for Vea1 3' UTR |
| OAS6663 | CTCAGACCCCCTCATGTCCT | fw Southern Blot Probe for Vea1 CDS |
| OAS1969 | TGCTATTGCTGCTGGAGGTG | rv Southern Blot Probe for Vea1 CDS |
| OAS6536 | GACGCTCAGGTCCAGTTCTC | fw primer in 3'UTR of Vea1 |
| OAS6525 | GGAGCATTACGGTCCCAACA | rv primer in 5'UTR of Vea1 |
| OAS5847 | GGGGACAAGTTTGTACAAAAAAGCAGGCTGTCAGACTGGATAGTTGAATGGTC | fw primer for P*_GAPDH_* + attB1 site |
| OAS6333 | GGACGAAACGAGTAAGCTCGTCGGCGGTCTGGGTGCCCTCGTGTTTTAGAGCTAGAAATAGCAAG | fw primer for 3’ fragment of CRISPR sgRNA cassette 1 for mCherry disruption |
| OAS7470 | GACGAGCTTACTCGTTTCGTCCTCACGGACTCATCAGGGCGGTGTTTTCTGGATATGGAT | rv primer for P*_GAPDH_* that incorporates mCherry protospacer 1 |

**Table S3: Strains used in this study**

| **Strain** | **Genotype** | **Description** |
| --- | --- | --- |
| G217B *ura5^-^* | *ura5^-^* | WU15, parental strain for Cas9 mediated genome modifications |
| HcB13 | *ura5^-^*, *URA5, Cas9* | Cas9 only expressing control strain (Cas9-1) |
| HcB14 | *ura5^-^*, *URA5, Cas9* | Cas9 only expressing control strain (Cas9-2) |
| HcB33 | *ura5^-^, ryp2^-^* | CRISPR/Cas9 generated *ryp2^-^*mutant |
| HcB34 | *ura5^-^, ryp2^-^* | CRISPR/Cas9 generated *ryp2^-^*mutant |
| HcB15 | *ura5^-^, sre1^-^* | CRISPR/Cas9 generated *sre1^-^*mutant (sre1-1) |
| HcB16 | *ura5^-^, sre1^-^* | CRISPR/Cas9 generated *sre1^-^*mutant (sre1-21) |
| HcB23 | *ura5^-^, sre1^-^, sid1^-^* | CRISPR/Cas9 generated *sre1^-^ sid1^-^ double* mutant |
| HcB24 | *ura5^-^, sre1^-^, sid1^-^* | CRISPR/Cas9 generated *sre1^-^ sid1^-^ double* mutant |
| HcB27 | *ura5^-^, sid1^-^* | CRISPR/Cas9 generated *sid1^-^*mutant |
| HcB28 | *ura5^-^, sid1^-^* | CRISPR/Cas9 generated *sid1^-^*mutant |
| HcB17 | *ura5^-^, sre1^-^, URA5, 5'UTR(p)::SRE1::CATB(t)* | Complemented *sre1^-^* mutant with episomal vector expressing *SRE1* under its native promoter |
| HcB18 | *ura5^-^, sre1^-^, URA5, 5'UTR(p)::SRE1::CATB(t)* | Complemented *sre1^-^* mutant with episomal vector expressing *SRE1* under its native promoter |
| HcB20 | *ura5^-^, sre1^-^, URA5, GAPDH(p)::SRE1::CATB(t)* | Complemented *sre1^-^* mutant with episomal vector expressing *SRE1* under constitutive GAPDH promoter |
| HcB21 | *ura5^-^, sre1^-^, URA5, GAPDH(p)::SRE1::CATB(t)* | Complemented *sre1^-^* mutant with episomal vector expressing *SRE1* under constitutive GAPDH promoter |
| HcKW104 | *ura5^-^, vea1∆* | CRISPR/Cas9 deletion mutant of *VEA1* |
| HcKW105 | *ura5^-^, vea1∆* | CRISPR/Cas9 deletion mutant of *VEA1* |
| mCherryHcG217B | ura5-/URA5, *zzz::T-DNA P_CBP1_::mCherry* | Integrated mCherry strain generated through *Agrobacterium*-mediated transformation (6) |

**Supplemental Figures**

**
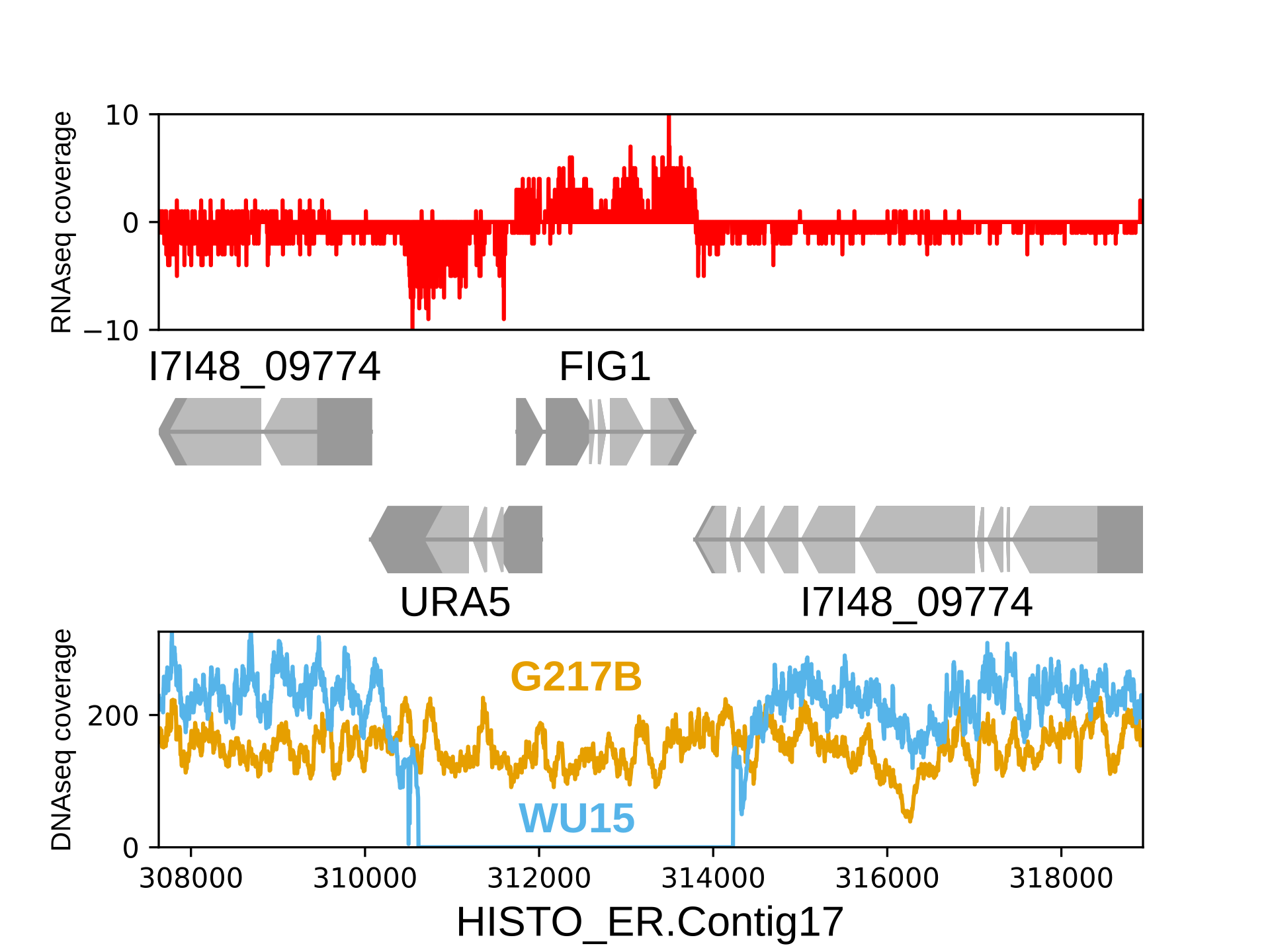
**

**Figure S1. Schematic indicating the URA5 deletion site in WU15.**Whole genome DNAseq coverage from G217B (7) and the WU15-derived strain G217B_ura5_old are plotted in orange and blue respectively.  RNAseq coverage (8) is plotted in red, and RNAseq derived gene models (8) are indicated between the two coverage plots.  *FIG1* is the adjacent gene to *URA5*. A clean break in the blue coverage plot indicates complete deletion of the *URA5* and *FIG1* CDS, and 3' truncation of the I714_09447 CDS, in WU15-derived strains.

**Figure S2.** **Deletion of *VEA1* CDS.** Restriction map of the *VEA1* wild-type and *vea1*∆ loci with cutting sites for Cas9-sgRNAs at the 5’- and 3’-end of the *VEA1* coding sequence. Restriction sites for *Bgl*II and the probe used for the Southern hybridization are shown. Genomic DNA was restricted with *Bgl*II and subjected to Southern blotting with the *VEA1* probe. The wild-type band is not present in the mutant isolates, confirming the deletion of *VEA1* in the mutants.

**Figure S3. Bioinformatics pipeline for variant assessment in the genomes of sequenced isolates as described in text.**

**Supplemental References**

1. Nødvig CS, Nielsen JB, Kogle ME, Mortensen UH. 2015. A CRISPR-Cas9 System for Genetic Engineering of Filamentous Fungi. PLoS ONE 10:e0133085.

2. Kujoth GC, Sullivan TD, Merkhofer R, Lee T-J, Wang H, Brandhorst T, Wüthrich M, Klein BS. 2018. CRISPR/Cas9-Mediated Gene Disruption Reveals the Importance of Zinc Metabolism for Fitness of the Dimorphic Fungal Pathogen Blastomyces dermatitidis. mBio 9:e00412-18.

3. Rodriguez L, Voorhies M, Gilmore S, Beyhan S, Myint A, Sil A. 2019. Opposing signaling pathways regulate morphology in response to temperature in the fungal pathogen Histoplasma capsulatum. PLoS Biol 17:e3000168.

4. Rodriguez L, Voorhies M, Gilmore S, Beyhan S, Myint A, Sil A. 2023. Retraction: Opposing signaling pathways regulate morphology in response to temperature in the fungal pathogen Histoplasma capsulatum. PLoS Biol 21:e3002060.

5. Beyhan S, Gutierrez M, Voorhies M, Sil A. 2013. A Temperature-Responsive Network Links Cell Shape and Virulence Traits in a Primary Fungal Pathogen. PLoS Biology 11:e1001614.

6. Van Prooyen N, Henderson CA, Hocking Murray D, Sil A. 2016. CD103+ Conventional Dendritic Cells Are Critical for TLR7/9-Dependent Host Defense against Histoplasma capsulatum, an Endemic Fungal Pathogen of Humans. PLoS Pathogens 12:e1005749.

7. Sepúlveda VE, Márquez R, Turissini DA, Goldman WE, Matute DR. 2017. Genome Sequences Reveal Cryptic Speciation in the Human Pathogen Histoplasma capsulatum. mBio 8.

8. Gilmore SA, Voorhies M, Gebhart D, Sil A. 2015. Genome-Wide Reprogramming of Transcript Architecture by Temperature Specifies the Developmental States of the Human Pathogen Histoplasma. PLoS Genetics 11:e1005395.
